# The updated mouse universal genotyping array bioinformatic pipeline improves genetic QC in laboratory mice

**DOI:** 10.1093/g3journal/jkae193

**Published:** 2024-09-13

**Authors:** Matthew W Blanchard, John Sebastian Sigmon, Jennifer Brennan, Chidima Ahulamibe, Michelle E Allen, Sam Ardery, Ralph S Baric, Timothy A Bell, Joseph Farrington, Dominic Ciavatta, Marta C Cruz Cisneros, Madison Drushal, Martin T Ferris, Rebecca C Fry, Christiann Gaines, Bin Gu, Mark T Heise, Pablo Hock, Richard Austin Hodges, Mia Hulgin, Tal Kafri, Rachel M Lynch, Terry Magnuson, Darla R Miller, Caroline E Y Murphy, David Truong Nguyen, Kelsey E Noll, Megan K Proulx, Christopher M Sassetti, Sarah A Schoenrock, Ginger D Shaw, Jeremy M Simon, Clare M Smith, Miroslav Styblo, Lisa M Tarantino, Joyce Woo, Fernando Pardo Manuel de Villena

**Affiliations:** Department of Genetics, University of North Carolina, Chapel Hill, NC 27599, USA; Mutant Mouse Resource and Research Center, Department of Genetics, University of North Carolina, Chapel Hill, NC 27599, USA; Department of Computer Science, University of North Carolina, Chapel Hill, NC 27599, USA; Mutant Mouse Resource and Research Center, Department of Genetics, University of North Carolina, Chapel Hill, NC 27599, USA; Mutant Mouse Resource and Research Center, Department of Genetics, University of North Carolina, Chapel Hill, NC 27599, USA; Department of Genetics, University of North Carolina, Chapel Hill, NC 27599, USA; Department of Genetics, University of North Carolina, Chapel Hill, NC 27599, USA; Systems Genetics Core Facility, Department of Genetics, University of North Carolina, Chapel Hill, NC 27599, USA; Genetics and Molecular Biology Curriculum, Department of Genetics, University of North Carolina, Chapel Hill, NC 27599, USA; Department of Epidemiology, Gillings School of Public Health, University of North Carolina, Chapel Hill, NC 27599, USA; Department of Genetics, University of North Carolina, Chapel Hill, NC 27599, USA; Department of Genetics, University of North Carolina, Chapel Hill, NC 27599, USA; Department of Genetics, University of North Carolina, Chapel Hill, NC 27599, USA; Mutant Mouse Resource and Research Center, Department of Genetics, University of North Carolina, Chapel Hill, NC 27599, USA; Department of Genetics, University of North Carolina, Chapel Hill, NC 27599, USA; Genetics and Molecular Biology Curriculum, Department of Genetics, University of North Carolina, Chapel Hill, NC 27599, USA; Department of Genetics, University of North Carolina, Chapel Hill, NC 27599, USA; Systems Genetics Core Facility, Department of Genetics, University of North Carolina, Chapel Hill, NC 27599, USA; Department of Genetics, University of North Carolina, Chapel Hill, NC 27599, USA; Systems Genetics Core Facility, Department of Genetics, University of North Carolina, Chapel Hill, NC 27599, USA; Department of Environmental Sciences and Engineering, Gillings School of Public Health, University of North Carolina, Chapel Hill, NC 27599, USA; Department of Genetics, University of North Carolina, Chapel Hill, NC 27599, USA; Department of Neuroscience, The Ohio State University, Columbus, OH 43210, USA; Department of Genetics, University of North Carolina, Chapel Hill, NC 27599, USA; Department of Genetics, University of North Carolina, Chapel Hill, NC 27599, USA; Department of Genetics, University of North Carolina, Chapel Hill, NC 27599, USA; Systems Genetics Core Facility, Department of Genetics, University of North Carolina, Chapel Hill, NC 27599, USA; Systems Genetics Core Facility, Department of Genetics, University of North Carolina, Chapel Hill, NC 27599, USA; Department of Microbiology and Immunology, University of North Carolina, Chapel Hill, NC 27599, USA; Department of Genetics, University of North Carolina, Chapel Hill, NC 27599, USA; Department of Genetics, University of North Carolina, Chapel Hill, NC 27599, USA; Mutant Mouse Resource and Research Center, Department of Genetics, University of North Carolina, Chapel Hill, NC 27599, USA; Lineberger Comprehensive Cancer Center, University of North Carolina, Chapel Hill, NC 27599, USA; Department of Genetics, University of North Carolina, Chapel Hill, NC 27599, USA; Department of Genetics, University of North Carolina, Chapel Hill, NC 27599, USA; Department of Genetics, University of North Carolina, Chapel Hill, NC 27599, USA; Department of Microbiology and Immunology, University of North Carolina, Chapel Hill, NC 27599, USA; Department of Microbiology, UMass Chan Medical School, Worchester, MA 01655, USA; Department of Microbiology, UMass Chan Medical School, Worchester, MA 01655, USA; Department of Genetics, University of North Carolina, Chapel Hill, NC 27599, USA; Department of Genetics, University of North Carolina, Chapel Hill, NC 27599, USA; Department of Genetics, University of North Carolina, Chapel Hill, NC 27599, USA; Department of Molecular Genetics and Microbiology, Duke University, Durham, NC 27710, USA; Department of Nutrition, Gillings School of Public Health, University of North Carolina, Chapel Hill, NC 27599, USA; Department of Genetics, University of North Carolina, Chapel Hill, NC 27599, USA; Department of Genetics, University of North Carolina, Chapel Hill, NC 27599, USA; Department of Genetics, University of North Carolina, Chapel Hill, NC 27599, USA; Mutant Mouse Resource and Research Center, Department of Genetics, University of North Carolina, Chapel Hill, NC 27599, USA; Systems Genetics Core Facility, Department of Genetics, University of North Carolina, Chapel Hill, NC 27599, USA; Lineberger Comprehensive Cancer Center, University of North Carolina, Chapel Hill, NC 27599, USA

**Keywords:** genetic QC, genetic background, substrains, chromosomal sex, genetic constructs, diagnostic SNPs, microarrays, inbred strains

## Abstract

The MiniMUGA genotyping array is a popular tool for genetic quality control of laboratory mice and genotyping samples from most experimental crosses involving laboratory strains, particularly for reduced complexity crosses. The content of the production version of the MiniMUGA array is fixed; however, there is the opportunity to improve the array's performance and the associated report's usefulness by leveraging thousands of samples genotyped since the initial description of MiniMUGA. Here, we report our efforts to update and improve marker annotation, increase the number and the reliability of the consensus genotypes for classical inbred strains and substrains, and increase the number of constructs reliably detected with MiniMUGA. In addition, we have implemented key changes in the informatics pipeline to identify and quantify the contribution of specific genetic backgrounds to the makeup of a given sample, remove arbitrary thresholds, include the Y Chromosome and mitochondrial genome in the ideogram, and improve robust detection of the presence of commercially available substrains based on diagnostic alleles. Finally, we have updated the layout of the report to simplify the interpretation and completeness of the analysis and added a section summarizing the ideogram in table format. These changes will be of general interest to the mouse research community and will be instrumental in our goal of improving the rigor and reproducibility of mouse-based biomedical research.

## Introduction

Genotyping arrays have been a staple in mouse research for more than 2 decades and have been successfully adopted for genetic quality control (GQC) and colony maintenance (Petkov et al. 2004; Yang et al. 2009, 2011; Morgan et al. 2015; Andrews et al. 2021; Amos-Landgraf et al. 2022). Five years after its introduction, the MiniMUGA array has been used for genotyping over 40,000 mouse samples, and the manuscript describing the array and its capabilities has been cited widely (Sigmon et al. 2020; Birling et al. 2022; Bourdon and Montagutelli 2022; Smith et al. 2022; Yoshiki et al. 2022; Teboul et al. 2024). Part of MiniMUGA's success is due to its unique characteristics, including discrimination between commercial substrains, robust chromosomal sex determination, and detection of commonly used constructs. In addition, a key advantage of MiniMUGA is the inclusion of a descriptive summary report of the sample that accompanies cost-effective genotypes. This has made MiniMUGA an attractive tool for organizations charged with genetic GQC of important collections such as the Mutant Mouse Resource and Research Centers (Amos-Landgraf et al. 2022, https://www.mmrrc.org/), and of large and complex breeding programs like the collaborative cross (CC) (https://csbio.unc.edu/CCstatus/index.py). Despite these successes and the fact that the MiniMUGA content is fixed (i.e. production array), several limitations of the original analysis pipeline can be addressed now. These limitations were caused, in part, by the modest number and type of samples genotyped in the production version of the array at the time (< 1,500 out of almost 8,000 used in that publication) and used for the initial marker annotation and bioinformatic pipeline validation (Sigmon et al. 2020). This negatively affected marker annotation, from marker performance to diagnostic information. Additional limitations included the exclusive use of a “greedy” algorithm for determining the contribution of primary and secondary genetic backgrounds, the exclusion of Y chromosome and mitochondria from the ideogram, the use of an overly restrictive threshold for the level of sample inbreeding required to run the informatics pipeline to completion and the limited number of biological replicates used in the creation of consensus genotypes for substrains.

Here, we report our efforts to improve marker annotation based on over 8,500 samples genotyped in the final array, increase the number of biological replicates used to generate consensus genotypes, improvement in the analysis pipeline for background selection, ideogram content, construct detection, and a new section that summarizes the genome complement in the form of diplotype intervals. These changes improve the rigor of the analysis and make the report useful for noninbred samples such as experimental crosses and partially congenic lines. We are committed to continuing this cycle of improvements in the future.

## Materials and methods

### Mice

We used 8,559 mouse DNA samples to analyze and annotate SNP marker performance. Samples included in this set were of excellent genotyping quality on the production version of the MiniMUGA array (defined by Sigmon et al. 2020). In addition, we selected samples with varying levels of inbreeding and different genetic backgrounds to increase the likelihood of capturing all 3 possible genotypes at each SNP marker (reference, alternative, and heterozygous; hereafter ref, alt, and het, respectively). We used 1,642 mouse DNA samples from 242 distinct inbred strains to create new consensus reference genotypes. Transnetyx and Neogen provided 196 samples to add consensus genotypes for 10 strains and to increase the number of biological replicates in 12 strains with consensus reported in the original description of MiniMUGA. We used 6 mice provided by the MMRRC as positive controls for the flipase (Flp) construct and 4 mice included in the initial description of the array as positive controls for the chicken HS4 insulator (cHS4) construct. Supplementary Table 1 lists the origin of the samples included in the SNP marker performance annotation. If the sample was used in the consensus genotypes, it lists the corresponding strain name; otherwise, it is annotated as N/A. It also states if the sample was used as positive or negative controls for Flp or cHS4 constructs; otherwise, it is listed as an “experimental sample”. Samples are categorized as inbred or outbred. Finally, samples are identified with a random 6-digit ID, and a reference is provided for previously reported samples.

An additional 16,123 samples genotyped by the Pardo Manuel de Villena (PMV) lab at the University of North Carolina (UNC) were used for manual curation and validation of our results.

### Marker annotation

The updated marker annotation is provided in Supplementary Table 2. This file includes marker annotations for the fields shown in Supplementary Table 3 (fields in bold are new or have updated information)

### Consensus genotypes

We used 1,642 mouse DNA samples from 242 distinct inbred strains to create new consensus reference genotypes. The updated consensus genotypes are provided in Supplementary Table 4. This table lists inbred strains for which we have created consensus genotypes. The table lists the number of biological replicates used to generate the consensus in parenthesis after the strain name.

### Synthetic backgrounds

For each of the 146 pairs of related substrains, we generated synthetic consensus in silico backgrounds. These are added to the consensus genotypes used in primary and secondary background determination and contribution. To generate a synthetic background, we compare the consensus genotypes at each SNP marker for a pair of substrains. If the substrain genotypes are the same call (A, T, G, C, or H), the resulting synthetic background call is that call. If the calls are different, the resulting synthetic call is an H. Note that this is the same as assigning H calls to the synthetic background at every marker annotated as diagnostic for either of the substrains in the pair.

### Strain annotation

Classical inbred strains have been assigned to one of 12 groups based on their name and origin (Supplementary Table 5). The rows in Supplementary Table 5 list all classical strains, while the columns list the strain groups used to determine the diagnostic potential of SNPs and a common outgroup with all classical inbred strains without diagnostic SNPs. Numbers in each cell are the number of diagnostic SNPs followed by the number of partially diagnostic SNPs, separated by a forward slash. N/A is not applicable because, by definition, they cannot have diagnostic SNPs. Blank cells should be ignored.

## Results

This update takes advantage of the large increase in the number of DNA samples genotyped with the production array since our initial description of the MiniMUGA platform (Sigmon et al. 2020) to (1) update the annotation of markers, (2) generate higher quality consensus genotypes for a larger number of inbred strains, and (3) update the content and layout of the report.

### Updated annotation of SNP marker performance

To address some of the limitations of the MiniMUGA pipeline, we reassessed the performance of the 10,819 SNP markers in the array and used that information to improve their performance annotation. For this task, we used a set of 8,559 excellent-quality samples genotyped with the production version of the array (‘*Materials and Methods*’; Supplementary Table 1). This set includes 1,943 inbred samples (or samples with very low levels of residual heterozygosity) and 6,616 outbred samples, including 535 F1 hybrid mice. Most outbred samples in this set are experimental backcrosses or intercrosses. Both sexes are similarly represented, and there are examples of sex chromosome aneuploids [4,239 XX, 4,276 XY, 36 XO, and 8 XXY classified based on our described method (Sigmon et al. 2020)]. The representation of homozygous and heterozygous calls, as well as both sexes, is critical for improved annotation of SNP marker performance.

**Fig. 1.**
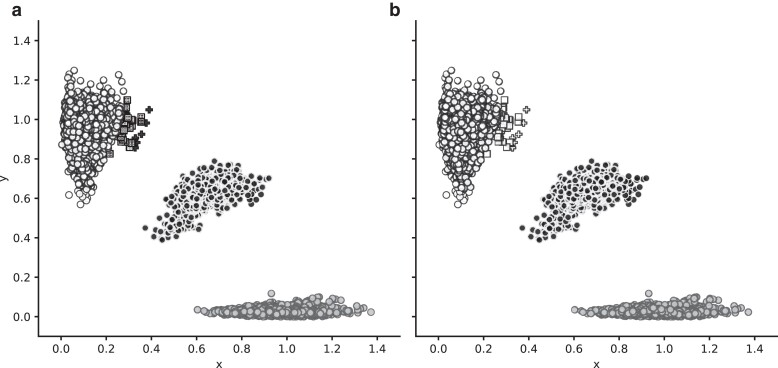
Intensity plot for Tier 1A marker gUNC11564970 (rs30612149). Panel A shows the genotype calls for 8,801 samples with the original clustering parameters and panel B shows the genotype calls for the same samples after reclustering. Each sample is shown as a circle, square, or cross. Fill pattern denotes genotype calls: solid gray, homozygous reference; solid white, homozygous alternate; solid black, heterozygous; and hatched markers, no genotype call. Shape denotes whether a sample genotype is the same (circles) or has changed from panels A to B (squares and crosses). Note that six samples shown as black crosses in panel A were originally called heterozygous incorrectly (they are in fact homozygous alternate). Fifty-six samples initially had no genotype call (hatched boxes in panel A), and these miscalls have been corrected in panel B using new clustering parameters.

We generated individual scatter plots for each SNP marker, plotting the normalized intensities for the performance sample set, using distinct colors to visualize the 3 standard genotype calls: ref, alt, and het, plus no calls (Fig. 1, Supplementary Fig. 1). As expected, the allele intensities are grouped into 3 distinct clusters representing the 3 standard calls (ref, alt, and het) for most markers. We identified 791 markers where at least 1 allele cluster included multiple different genotype calls due to incorrect genotype-calling software tuning (Fig. 1). We used the allele intensity plots to set new cluster boundaries and recalled all 8,559 samples at these 791 markers. We then compared plots and genotype calls to identify SNPs where the new cluster boundaries produce more consistent genotype calls (fewer clusters with inconsistent genotype calls) and fewer no calls overall. The new cluster boundaries were updated for 756 markers (733 markers from training array content and 23 additional markers from production array content) where there is an improvement in the congruency of the genotype calls (compare a and b in Fig. 1). Users should expect some discrepancies between the new and original genotype calls at these markers, given that the cluster boundaries have changed.

The next step was to address the performance of individual SNPs after reclustering based on the number of clusters, the discrimination between clusters, and the level of consistency of genotype calls within clusters in the performance sample set. We use these metrics to define a new performance annotation called Tier 2022. First, we divided markers into 3 broad classes (called Tiers): Tier 1 makers have 3 distinct clusters at the expected locations for reference, alternate, and heterozygous genotype calls for autosomal and X chromosome markers, and 2 distinct reference and alternate clusters at Y chromosome and mitochondrial markers. Tier 2 markers have an additional cluster of no calls at or near the intensity plot origin (these samples fail to produce a signal for either allele). This cluster is predicted to be due to off-target variants within the 50 bases of the array probe in one or more haplotype(s) (Didion et al. 2012, Supplementary Fig. 1). We used whole-genome sequencing (WGS) to confirm this prediction in a small set of cases (data not shown). Tier 4 markers exhibit no recognizable intensity clustering patterns (or form only one cluster) and fail to produce reliable genotype calls in the performance sample set. These markers should always be excluded from all analyses. The second level of classification applies to Tier 1 and 2 markers and divides them into 3 additional classes: Tiers A, B, and C. Tier A markers have the highest performance within a tier: the 3 genotype clusters are distinct, there are no inconsistent genotype calls within these clusters, and they have no or very few N calls, excluding the cluster of N calls at the plot origin for Tier 2 markers. Tiers B and C have decreasing performance levels, with lower discrimination between clusters, an increasing number of inconsistent genotype calls within one or multiple clusters, and an increasing number of N calls. There is no hard boundary between Tiers B and C as the performance depends on the haplotype present at the locus.

Table 1 summarizes the number of markers classified in each Tier. Tier 1A markers perform best across all samples and comprise 82.5% (8,929) of all SNP markers in the array. In the sample report, only Tier 1A markers are used to identify and quantify the contribution of the primary and secondary backgrounds and to determine the level of inbreeding. Although Tiers 1B, 2A, and 2B may perform well in many genetic backgrounds, they are not completely reliable and may produce incorrect genotype calls in some backgrounds. For example, for Tiers 2A and 2B markers, samples that are heterozygous for a combination of the off-target variant and any of the 2 standard alleles will genotype as homozygous in most cases (Didion et al. 2012). Finally, we manually downgraded the performance annotation of specific makers if we observed incorrect genotype calls in samples with known and defined genetic backgrounds (inbred, F1, and F2 mice).

**Table 1.** MiniMUGA SNP marker counts—grouped by Tier 2022 and diagnostic capability.

	1A	2A	1B	2B	1C	2C	4	Total
Diagnostic	3098 95.2%	107 3.3%	43 1.3%	6 0.2%	0	0	0	3254
Not Diagnostic	5831 77.1%	184 2.4%	152 2.0%	43 0.6%	209 2.8%	28 0.4%	1118 14.8%	7,565
Total	8929 82.5%	291 2.7%	195 1.8%	49 0.5%	209 1.9%	28 0.3%	1118 10.3%	10,819

Each cell in the table displays the number of markers with a given Tier and diagnostic capability, followed by the percentage of the row total this represents in parentheses.

### Expanded detection of genetic constructs

**Fig. 2.**
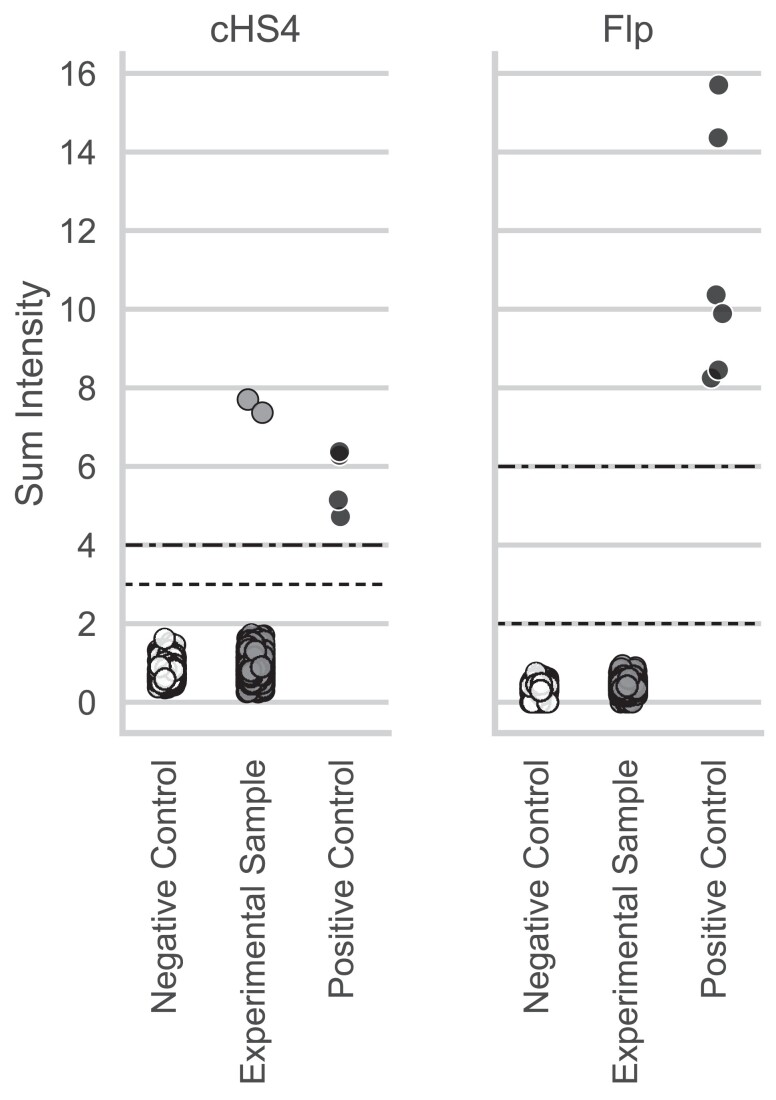
Detection of two new genetic constructs validated in MiniMUGA. Chicken HS4 insulator (cHS4) and Flippase (Flp). For each construct, individual samples are plotted as dots with vertical positions reflecting the sum of the intensity values for the corresponding construct markers in that sample. The samples are classified as negative controls (left, open circles), experimental (center, grey circles), and positive controls (right, black circles). The dashed horizontal lines represent data-driven thresholds discriminating between the presence or absence of each construct. Samples below the simple dashed line are considered negative, samples above the composite dotted-dashed line are considered positive, and samples in between the dashed lines are considered questionable.

We annotated 14 probes (Supplementary Fig. 2) which detected 2 additional constructs: cHS4 and Flippase (Flp). Figure 2 shows the aggregate performance of these probes in negative controls, positive controls, and experimental samples. Thresholds for presence and absence were determined as previously described for other constructs by minimizing the number of experimental samples with questionable presence of the corresponding construct while keeping positive and negative controls fully concordant (Sigmon et al. 2020).

### Improved annotation of diagnostic SNPs and homoplasy

A unique feature of MiniMUGA is the ability to discriminate between closely related substrains based on SNPs with diagnostic alleles. These are variants in which the diagnostic allele arose in the inbred ancestor of one or more but not all substrains. Because these SNPs were identified through WGS of a single mouse from the relevant substrain, the frequency of the diagnostic allele in that and related substrains is unknown a priori. A complicating factor in defining diagnosticity is that for some of these SNPs, the diagnostic allele is present in other inbred strains either due to homoplasy (2 independent mutation events resulting in the same variant) or shared local ancestry. Discrimination between the 2 phenomena is considered in the ‘*Discussion*’. Here, it suffices to say that only nonhomoplasic SNPs and homoplasic SNPs where the minor allele is present only in a wild-derived strain(s) are annotated as diagnostic. Because most commonly used laboratory mice do not contain wild-derived backgrounds, annotating the second group as diagnostic is an acceptable compromise.

To identify diagnostic SNPs, we first classified the 94 classical inbred strains and substrains into one of 12 strain groups or a common outgroup of 23 classical inbred strains (see Supplementary Table 5). Strain groups include all substrains, and this classification was based on naming conventions and historical records. A given SNP is considered to have a diagnostic allele if its minor allele segregates in a strain group and is absent in the 11 other strain groups and common outgroup. For these markers, the set of substrains in which the minor (now annotated as diagnostic) allele is observed is called its diagnostic class.

Diagnostic alleles observed in some but not all constituent samples used to create the genotype consensus for a given substrain are annotated as “partially diagnostic.” Note that SNPs with partially diagnostic alleles are typically not shared between substrains. SNPs with partially diagnostic alleles are reported as heterozygous (or H) in the consensus for the substrain they segregate (Supplementary Table 4).

By design (Supplementary Fig. 3), there is an overrepresentation (*n* = 3,254) of SNPs with diagnostic alleles in MiniMUGA to ensure that it can discriminate between as many substrains for which we had WGS at the time of the array design as possible. The performance of these SNPs is, on average, better than nondiagnostic markers. For example, 95.2% of markers with diagnostic alleles are in Tier 1A vs 77.1% of nondiagnostic markers. In addition, there are no SNPs with diagnostic alleles in Tier 4 vs 14.8% of nondiagnostic markers (Table 1). In conclusion, the presence of diagnostic alleles in a sample is reliable evidence for the contribution of the corresponding substrain(s) to the sample genome.

In this update, we have added new annotations to Supplementary Table 2 that identify diagnostic SNPs with homoplasy in a wild-derived strain and homoplasic SNPs with overwhelming evidence of a recent and independent mutation arising in a substrain that is not annotated as diagnostic because the allele is also present in one or more unrelated classical inbred strains (see ‘*Discussion*’, Supplementary Fig. 3).

### Expansion and update of consensus genotypes for inbred strains

We created new consensus genotypes for 242 inbred strains based on 1,642 samples (only 556 samples were used to create the previous consensus genotypes). All samples in the consensus set were curated to ensure they were pure representatives of the corresponding inbred strain. This consensus set includes 10 additional classical inbred strains (BALB/cAnNTac, BALB/cAnNRj, BALB/cByJRj, BALB/cByJJic, BALB/cJRj, C57BL/6JJicTac, CBA/CaJ, DBA/1JBomTac, DBA/1Rj, and SJL/JCrNTac) and genotypes for the 63 CC strains currently available from the Systems Genetics Core Facility (SGCF)/MMRRC–UNC.

Consensus genotype calls are a representation of the strain or substrain genotypes. The calls are generated according to previously described rules (Sigmon et al. 2020), with one notable change: SNPs with partially diagnostic alleles previously annotated as a lower-case diagnostic allele call are now annotated as H in the corresponding substrain. This H call indicates that the diagnostic allele is segregating in a substrain. Therefore, samples from this substrain may have any of the 3 possible genotypes at those markers. This change in the rule better represents the possible genotypes observed and simplifies the genome analysis algorithm.

**Fig. 3.**
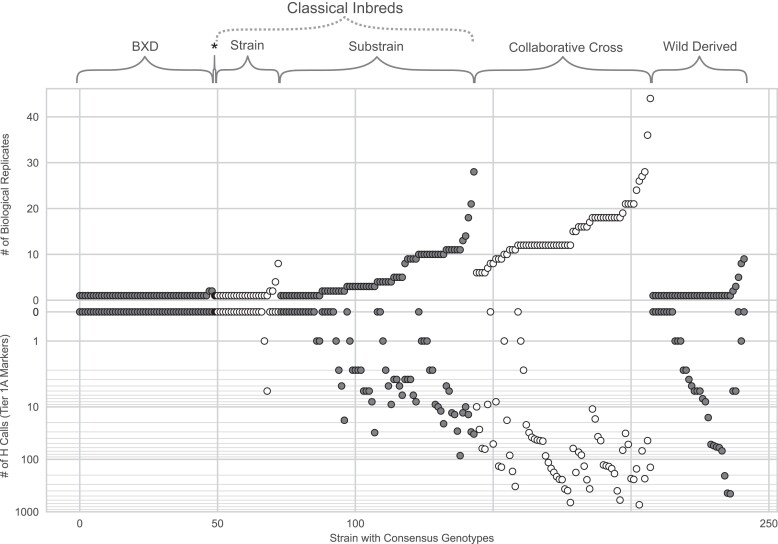
Number of biological replicates and heterozygous calls in the consensus genotypes in 242 inbred strains. Each dot represents an inbred strain from one of six strain types, grouped. Briefly, the asterisk denotes NOR/LtJ (a recombinant inbred strain derived from NOD/LtJ and C57BLKS/J). Supplementary Table 4 lists the full list of names of the strains represented in this figure. The upper *y*-axis reflects the number of biological replicates per consensus strain. The lower *y*-axis is the number of H calls per consensus strain, in an inverted log10 scale. Strains are presented in ascending order based on the number of biological replicates within the strain type. Only Tier 1A markers are included in the analysis.

The accuracy of the consensus depends on genotyping quality, the number of biological replicates (we define biological replicates as distinct mice from a given strain or substrain) included, and how comprehensive the sampling was in terms of dates and pedigree for each strain. We used all biological replicates (*n* = 1,521) and a small subset (*n* = 121) of technical replicates to generate the consensus genotypes. A maximum of one technical replicate per biological replicate was included. Figure 3 and Table 2 show the distribution of the number of biological replicates per strain, grouped by strain type. Figure 3 also shows the number of consensus H calls per strain below the *X*-axis. The average number of biological replicates varies among the different types of strains, and it reflects our goal to increase replicates in strains where we expect to detect true heterozygosity with MiniMUGA. CC strains have the highest average number of biological replicates (*n* = 15.06) because the consensus aims to capture regions of residual heterozygosity in each strain by including all male and female breeders alive for each strain in the SGCF in 2020. The type with the second highest number of replicates is composed of the 71 classical inbred substrains (*n* = 5.83). In this group, the high number of biological replicates is critical to determine whether diagnostic SNPs segregate within a substrain and thus should be annotated as partially diagnostic for the relevant substrain(s). Other classical strains (*n* = 23), wild-derived strains (*n* = 34), and finally, the strains in the BXD recombinant inbred panel have a lower number of replicates. A priori, these last 3 groups of inbred strains should have no H calls for Tier 1A markers. However, H calls can represent rare miscalls due to off-target variants (Didion et al. 2012). These are particularly prevalent in wild-derived strains (note that CC strains are derived from 8 founders, including 3 wild-derived strains).

**Table 2.** Constituent samples per consensus strain, organized by strain type, replicate type, and sex.

		Biological replicates			Male biological replicates			Total samples		
	strains	min	max	mean	min	max	mean	min	max	mean
Classical inbred—Substrain	71	1	28	5.8	0	15	3.3	1	29	6.9
Classical inbred—Strain	23	1	8	1.5	0	5	1.2	1	10	2.5
Wild derived	34	1	9	1.7	0	5	1.4	1	9	2.2
Collaborative cross	64	6	44	15.0	2	13	5.1	6	44	15.0
BXD	49	1	2	1.0	0	0	0.0	1	2	1.0
Other recombinant inbred	1	1	1	1.0	1	1	1.0	2	2	2.0

The table presents the total number of strains per strain type, the range and mean number of biological replicates per strain for each strain type, the range and mean number of male replicates per strain for each strain type, and the range and mean number of total replicates per strain for each strain type.

### Changes in the analysis pipeline and report layout

There are 5 main updates in the analysis pipeline and/or report presentation (Supplementary Fig. 4): (1) updated pipeline for genetic background determination in samples with more than one genetic background; (2) changes in the inbreeding estimation; (3) inclusion of the Y Chromosome and mitochondria in the ideogram; (4) specification of the “Minimal Strain Sets Explaining All Diagnostic Classes”; and (5) the addition of a table with diplotype intervals.

### Determination of global genetic background

The genome analysis section summarizes the global genetic background, showing the contribution of the primary, secondary, and further unexplained backgrounds, each as a percentage of the genome. This global summary complements the local information shown in the ideogram. The primary and secondary backgrounds are determined by the greedy algorithm described previously (Sigmon et al. 2020) with the following changes: (1) Background analysis now involves only the highest quality markers (Tier 1A); (2) The set of potential background strains now includes several synthetic backgrounds composed of pairs of related substrains generated in silico (see ‘*Materials and Methods*’). These were created specifically to enable the pipeline to work equally well for samples whose genetic background includes 2 related substrains plus an additional third (or even a fourth) strain; (3) The default threshold for clustering is set at 2 informative markers with consistent genetic background and zygosity (i.e. diplotype) per 2 Mb; (4) The requirement for samples to have less than a threshold level of heterozygosity has been removed and thus reports are created for most samples including F1 hybrids and F2 samples. As a result, the updated pipeline produces a complete report at more than twice the rate of the original (only 11,767 samples out of 26,245 (44.8%) in our database would have generated a full report with the original pipeline); and (5) We have lowered the threshold for the minimum fraction of the genome that must be explained to produce a report to 97.5% percent (was 99.8% previously).

In cases where more than one genetic background is necessary to explain a sample, we only use Tier 1A markers that are informative among the primary background, secondary background, and sample genotypes. We use the genome positions of these informative SNPs to identify contiguous regions in the sample genome with the same diplotype. We extend the proximal and distal cluster boundaries to the midpoint for the intervals between clusters with different diplotypes. For the most proximal and distal clusters, we extend them to the start and end of the corresponding chromosome. The resulting set of Diplotype Intervals is used: (1) to construct the Ideogram presented in the Local Genetic Background section of the report, (2) to estimate the contribution of the primary, secondary, and unexplained backgrounds presented in the Global Genetic Background, and (3) to calculate the overall Level of Inbreeding reported for the sample. This updated method of estimating the contribution of each genetic background using physical distance (in megabases) is a major improvement over the original pipeline. Specifically, the initial pipeline overestimated the contribution of the primary background to the sample genome by an average of 18% (range, 0–48%) (Supplementary Table 6) because it included noninformative homozygous markers in the estimation. It also underestimated the contribution of the secondary background to the sample genome by an average of 8.6% in homozygosity and heterozygosity (ranges, 0–27 and 0–33%, respectively). The contribution of any unexplained background to the genome was underestimated by 0.9% on average (0–7%). Error in the estimated contribution to genetic backgrounds in the original pipeline was proportional to the amount of nonprimary background present in a sample. In summary, the updated pipeline provides more accurate estimates than the original pipeline, particularly in samples with similar contributions of 2 genetic backgrounds, including F2 and F1 hybrids.

### Changes in the inbreeding estimate

The inbreeding estimate is based on the percentage of the nuclear genome, excluding the Y chromosome, which is homozygous (or hemizygous for males) for primary, secondary, and unexplained backgrounds. In contrast to the previous estimate, this update is far less dependent on the number and density of informative SNPs for a given pair of backgrounds. This is especially relevant for samples derived from 2 closely related substrains (Supplementary Fig. 5).

### Representation of the local genetic background in the ideogram

The report (Supplementary Fig. 4) shows the local genetic makeup of the sample in a visualization of the genome known as an ideogram. This is as described in the original paper (Sigmon et al. 2020), with the addition of the Y chromosome (shown as a single bar) and the mitochondria (shown as a torus). The genome regions shown in black represent the primary or majority genetic background. The regions shown in red represent the secondary or minority genetic background. Red and black in the same region indicate heterozygosity. Transitions between primary and secondary backgrounds are placed at the midpoint between flanking informative markers (see above). Regions shown in white are identical by descent. This means that MiniMUGA cannot distinguish between the primary and secondary backgrounds in these regions. As mentioned above, this is especially relevant for samples derived from 2 closely related substrains. The MiniMUGA sample report is optimized for reproducible mouse models involving only 1 or 2 genetic backgrounds. Any unexplained regions will be shown in gray if more than 2 backgrounds are present.

Markers with diagnostic alleles for the primary and secondary background are also shown in the ideogram. Markers that are diagnostic for the primary background are represented by triangles at the corresponding position on the left side of the chromosome. Similarly, markers that are diagnostic for the secondary background are represented by triangles on the right side of the chromosome. Black-filled triangles indicate the presence of the diagnostic allele for the primary background at the corresponding SNP in the sample. Red-filled triangles indicate the presence of the diagnostic allele for the secondary background at the corresponding SNP in the sample. Note that filled triangles do not imply homozygosity or heterozygosity for the diagnostic allele. Unfilled triangles indicate the absence of the diagnostic allele. Diagnostic markers shown as unfilled triangles can be inconsistent with the local diplotype shown in the ideogram due to the existence of SNPs with partially diagnostic alleles. For example, unfilled triangles can sometimes be found in pure representatives of the corresponding substrain and its derivatives.

### Reporting of the substrains detected with the diagnostic alleles

Utilizing the updated diagnostic annotations, we now report the number and zygosity of SNPs with diagnostic alleles for each of the 94 diagnostic classes (Supplementary Table 2) present in a sample. As previously discussed, a diagnostic class groups all substrains sharing specific diagnostic alleles and thus reflects the local ancestry of the substrains. The report programmatically determines the minimum set, or sets, of substrains required to explain all the diagnostic alleles present in a sample. This is presented as “*Minimal Strain Sets Explaining All Diagnostic Classes*” in the report. There are 4 important caveats to consider here. First, if multiple solutions are presented (multiple combinations of substrains), the user is encouraged to use external information to select the most likely solution, if available. Second, the minimal solution may not include all the genetic backgrounds present in a sample. Any combinations of substrains covering all diagnostic classes can be the “true” solution (but not the minimal). Third, the primary and/or secondary background reported in the genome analysis section is not required to cover the “Minimal Strain Sets Explaining All Diagnostic Classes” present in a sample. Genotypes at diagnostic SNPs are used in selecting primary and secondary backgrounds but are not given any special weight or consideration. Finally, we reiterate the point made in the original paper (Sigmon et al. 2020) that these markers are unreliable in mice with contributions from wild-derived strains.

### Addition of diplotype intervals

This new section presents the data in table format as shown in the ideogram. For each interval, the table provides the chromosome, start and end positions in GRCm38 genome coordinates, background (name(s) of the (sub)strain assigned to the interval), and zygosity.

## Discussion

We are keenly aware that since the public release of the MiniMUGA array, many users have struggled with and spend an inordinate amount of effort dealing with a few inconsistent and/or unexpected genotype calls in a sample (e.g. H calls in inbred samples, the presence of an unexpected diagnostic allele in “pure” sample, etc.). In addition, some samples failed to generate a complete report, and quantitative data for inbreeding and background contributions was, in some cases, misleading. To address these issues, we updated all SNP markers’ performance and diagnostic capability of the annotations, increased the number of biological replicates and strains for which we have consensus genotypes, and improved the informatic pipeline. Although the report provides many pieces of qualitative and quantitative information, the user should remember that the 2 most important sections are first the “Genome Analysis” section that provides the identity and contribution of primary and secondary backgrounds, the level of inbreeding, and an ideogram based on that information; and second the “Backgrounds Detected (Diagnostic Alleles)” that lists the substrains detected based on diagnostic alleles.

Regarding Tier annotation, reclustering for selected markers leads to better performance as measured by the level of consistent genotype calls between samples that carry the same allele. Importantly, some identical (or related) samples genotyped using the original and the new clustering parameters may have differing genotypes at some markers. Users should check whether these markers are annotated as reclustered in Supplementary Table 2. In these cases, genotype changes are likely due to the analytical pipeline rather than biological reasons. In general, the genotypes generated with the updated pipeline should be preferred.

In the future, adding new samples may upgrade some markers from low performance to a higher performance tier. In addition, we will continue to manually curate markers with inconsistent annotations (e.g. tier and diagnostic value) or subpar performance in specific samples. If the number of markers with annotation changes is significant, we will consider releasing a public update.

The updated consensus genotypes differ in 2 important aspects with respect to those described in the original paper. First, we now base our updated consensus on an expanded number of biological replicates to capture true heterozygosity better. This effort was focused on substrains from classical inbred strains and the CC population. Second, we added new substrains to our catalog. This will enable the identification of the correct genetic backgrounds of samples involving these substrains.

The presence and number of SNPs with diagnostic alleles are among the major features that distinguish MiniMUGA from other mouse genotyping arrays. Although we use a simple and unambiguous definition for SNPs with diagnostic alleles (they must arise in an inbred strain and discriminate between substrains of that strain), in practice, the identification, annotation, and use of SNPs with diagnostic alleles involves some uncertainties. First, by definition, SNPs with diagnostic alleles are very rare (from a few hundreds to a few thousands in a given substrain), and their identification requires deep WGS (in fact, low or medium coverage in WGS is typically not enough to identify the presence and zygosity of these SNPs in a sample with mixed background). The rarity is because these mutation events have occurred in the last 5 to 70 years, compared to all mutations that have accumulated over millions of years of evolution in the *Mus* lineage (Supplementary Fig. 3). Second, typically, only one or very few mice are sequenced and used to identify diagnostic variants. Because these are recent mutations in that substrain or in its recent ancestors, a priori, we do not know the allele frequency of these variants in the substrain, and we should expect many to be segregating. Furthermore, samples from that substrain that precede the mutation event will lack the diagnostic allele. Therefore, many SNPs with diagnostic alleles are expected to be partially diagnostic. A second complicating factor is that SNPs in which the minor allele is exclusively seen in the relevant substrain are unambiguously diagnostic; i.e. it is extremely unlikely that that allele will be found in any laboratory mouse that is unrelated to the relevant substrain because our survey of classical and wild-derived strains covers most of the lab mouse universe. However, for 295 (9%) annotated diagnostic SNPs, the diagnostic allele is also found in one or more wild-derived strains (Supplementary Table 2). The fact that these SNPs are diagnostic is not in doubt because they are segregating among substrains derived from fully inbred ancestors. The presence of the same allele (or an allele that is undistinguishable with Illumina technology, for example, A/G and A/C) in a wild-derived strain is evidence of homoplasy, and it is expected given the ample evolutionary times considered (Supplementary Fig. 3). As a rule, the reported presence of a substrain based on a single heterozygous call at a diagnostic SNP should be treated with caution. Future addition of new substrain samples may lead to annotation changes from fully diagnostic to partially diagnostic. The addition of new substrains may lead to new diagnostic class annotations.

Note that other mouse genotyping arrays sample variation proportionally to the phylogenetic history of the samples used for SNP selection, for example, the Mouse Diversity Array for general use in laboratory mice and previous Mouse Universal Genotyping Array (MUGA) iterations for genetic analysis in the CC (Yang et al. 2009; Morgan et al. 2015). In contrast, MiniMUGA was designed to magnify differences among the most closely related samples used in SNP selection (i.e. substrains). Panels A and B in Supplementary Fig. 3 show the same phylogenetic tree for a set of putative strains used in SNP selection. In panel A, the time scale is shown in natural years, while in panel B, time is shown in a log scale. The second panel provides a useful analogy for the relative representation of SNPs in MiniMUGA.

Regarding improvements in the informatic pipeline, the report uses only Tier 1A markers to determine the primary and secondary backgrounds. The report uses all Tier 1A, 1B, 2A, and 2B markers annotated as diagnostic in the Backgrounds Detected (Diagnostic Alleles) section. The selection of markers from different Tiers in these different analyses reflects that Tier 1A markers are reliable across all genetic backgrounds for local and global genome analysis. None of the 4 tiers listed above erroneously detect diagnostic alleles (false positives) in any sample tested.

Our general advice to those planning to use MiniMUGA genotype data for genetic mapping is to submit enough biological replicates from both sexes from the parental strains involved in the cross if the consensus is based on one or few biological replicates or may have fixed some truly segregating SNPs. We also recommend genotyping the corresponding F1 hybrids and using those genotypes to select informative markers with fully consistent genotypes in parentals and F1s. These will be mostly Tier 1A markers, but some Tier 1B, 2A, and 2B markers may be selected and useful for specific experimental crosses.

Including the mitochondria and the Y Chromosome in the ideogram was a long overdue improvement that will easily alert users of congenic strains, among others, of potential errors or limitations in their mice. When an ideogram has regions where the primary and secondary backgrounds are highly fractured (having frequent changes of genetic background and/or zygosity over a short genomic distance), as shown in Supplementary Fig. 6, the selected genetic backgrounds are likely incorrect, or there are present. Fracturing can also be localized, and the same conclusion applies. The report will provide a warning in the Summary section if fracturing is detected.

In conclusion, the updates in the MiniMUGA pipeline reported here reduce the impact of under-performing SNP markers, increase the reliability of the reported genetic backgrounds, provide a better estimation of background contribution and level of inbreeding, expand the universe of samples for which a full report is generated, and provide new information including the potential presence of 2 additional constructs and detailed diplotype intervals. We hope these changes are useful, and we welcome comments from the community regarding further enhancements. Nonexpert users may want to take advantage of a recently released short video tutorial that provides a 10-minute guide on how to interpret a MiniMUGA sample report (https://www.med.unc.edu/mmrrc/).

## Data Availability

All sample genotype and intensity data used in the supporting analyses and development of this manuscript have been archived at the University of North Carolina Dataverse (Cruz Cisneros et al. 2024, https://doi.org/10.15139/S3/YQLDUJ). Supplementary material available at figshare: https://doi.org/10.25387/g3.26311777.
